# Optimization of an *In Vitro* Colonic Fermentation: Insights into Flavan-3-ol Catabolism and Microbiota Modulation

**DOI:** 10.1021/acs.jafc.5c06932

**Published:** 2025-06-18

**Authors:** Nicole Tosi, Leonardo Mancabelli, Giulia Alessandri, Francesca Turroni, Marco Ventura, Daniele Del Rio, Pedro Mena, Letizia Bresciani

**Affiliations:** † Human Nutrition Unit, Department of Food & Drug, University of Parma, Via Volturno 39, 43125 Parma, Italy; ‡ Department of Medicine and Surgery, University of Parma, Via Gramsci 14, 43125 Parma, Italy; § Microbiome Research Hub, University of Parma, Parco Area delle Scienze 11A, 43124 Parma, Italy; ∥ Laboratory of Probiogenomics, Department of Chemistry, Life Sciences, and Environmental Sustainability, University of Parma, Parco Area delle Scienze 11A, 43124 Parma, Italy

**Keywords:** (−)-epicatechin, procyanidins, gut microbiota, colonic catabolism

## Abstract

*In vitro* fecal fermentation models are essential for studying gut microbiota-mediated metabolism of dietary flavan-3-ols. Current methodologies typically limit fermentation periods to 24 h, potentially overlooking the complete kinetics of catabolites. This study aims to extend *in vitro* fecal fermentation of dietary (poly)­phenols up to 48 h to improve the physiological relevance of the model. Fermentation dynamics were assessed through the simultaneous monitoring of polyphenol catabolites, pH, and microbiota composition. One flavan-3-ol monomer ((−)-epicatechin) and two oligomers (procyanidin B2 and procyanidin A2) were fermented using human fecal slurry. Fourteen catabolites were quantified at five time points, revealing that flavan-3-ol polymerization and procyanidin linkage influence bioaccessibility and catabolism. The extended fermentation provided a more complete view of flavan-3-ol metabolism, with stable pH (5–6) and unaffected microbial composition. Substrate-specific effects on microbial alpha diversity suggest distinct resilience patterns, and putative associations between microbial taxa and phenolic catabolites were identified. This study demonstrates that 48 h incubation maintains physiological relevance, capturing late-stage catabolites, making the colonic model more reliable, with significant implications for understanding the colonic fate of undigested dietary (poly)­phenols and the microorganisms possibly involved in their transformation.

## Graphical abstract

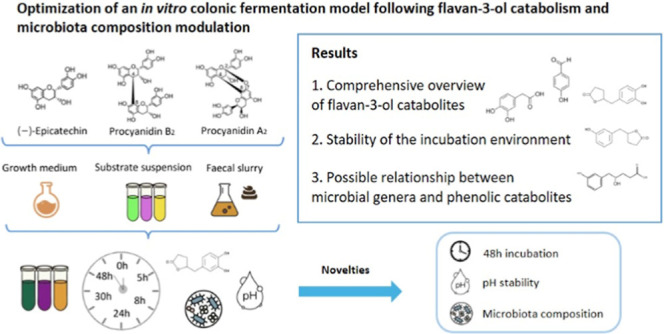

## 1 Introduction

Flavan-3-ols are the most consumed and ubiquitous flavonoids, with dark chocolate, green tea, nuts, and some fruits being the principal sources in the Western diet.^1,2^ From a structural point of view, flavan-3-ols are the most complex class of flavonoids, although they are the sole class that occurs in nature mainly in aglyconic form.^3,4^ Flavan-3-ols exist in a monomeric form or in an oligo/polymeric form, with the latter named proanthocyanidins or condensed tannins. The monomeric structure is characterized by two aromatic rings (A- and B-ring) and a pyran ring (C-ring) condensed with the A-ring. The C-ring is hydroxylated in position 3 and can be esterified with gallic acid, giving origin to galloylated flavan-3-ols. The A-ring presents two hydroxyl groups in positions 5 and 7, while hydroxylation of the B-ring varies giving origin to different types of monomers and proanthocyanidins: (1) (epi)­afzelechins, which have one hydroxyl group in position 4′, and their polymerization produces propelargonidins; (2) (epi)­catechins, presenting two hydroxyl groups in positions 3′ and 4′, giving origin to procyanidins through polymerization; (3) (epi)­gallocatechins, characterized by three hydroxyl groups in positions 3′, 4′, and 5′, respectively, and giving rise to prodelphinidins. (Epi)­catechins and procyanidins are the most common flavonoids in the diet, while (epi)­gallocatechins characterize green tea.^3–5^ Procyanidins are divided into two groups according to the number of linkages between monomeric units: B-type procyanidins are characterized by a C4–C8 or C4–C6 linkage. At the same time, A-type procyanidins present an additional ether linkage between the C2 of a monomer and the oxygen-bearing C5 or C7 in the A-ring of the adjacent unit.^5^ While B-type procyanidins are present in foods such as cocoa, fruits, nuts, and legumes, A-type procyanidins are less common and occur mainly in American cranberries and persimmon.^3–5^

After ingestion, only a minor part of the monomeric flavan-3-ols (30% of (epi)­catechins and 10% of (epi)­gallocatechins) and traces of proanthocyanidins are absorbed in the upper gastrointestinal tract. Unabsorbed flavan-3-ols reach the colon, where they are extensively catabolized by the gut microbiota, yielding a plethora of small microbial catabolites, which are finally absorbed and conjugated by phase II human enzymes in colonocytes and hepatocytes.^6^ The catabolic pathway of B-type procyanidins can follow four different routes: (1) interflavan cleavage, releasing (epi)­catechin monomers, (2) degradation of the lower unit, giving origin to phenyl-γ-valerolactones, (3) degradation of the upper unit, yielding 3′,4′-dihydroxyphenylacetic acids, and (4) simultaneous degradation of the upper and lower units, yielding 5-(2′,4′-dihydroxyphenyl)-2-ene-valeric acid.^5^ Monomeric units undergo C-ring opening, giving origin to diphenylpropan-2-ol derivatives and A-ring fission, yielding phenyl-γ-valerolactones, which can be hydrolyzed and converted into phenylvaleric acids.^7^ Diphenylpropan-2-ols, phenylvaleric acids, and phenyl-γ-valerolactones are peculiar flavan-3-ol catabolites,^5^ which are converted into small microbial catabolites through different steps of β- and/or α-oxidation of the side chain, generating (1) phenylpropanoic acids, (2) phenylacetic acids, and (3) benzoic acids and benzaldehydes, originated by the reduction of benzoic acids. Finally, benzene derivatives can originate from the decarboxylation of benzoic acids.^8^

Consumption of flavan-3-ols has been associated with risk reduction of several chronic diseases, including cardiometabolic and neurodegenerative diseases.^9–12^ Microbial catabolites and their phase II conjugates are the circulating molecules that can potentially have beneficial effects on different cellular and tissue targets.^13–16^ The native structure of flavan-3-ols, including the degree of polymerization and the type of subunit linkages, can influence the type and amount of gut microbiota catabolites, having an impact on health effects.^17^ Polymerization of flavan-3-ols increases the steric hindrance of the molecule, limiting access to the C-ring by the gut microbiota, with a reduction of bioaccessibility and delayed catabolism.^17–20^ A reduction of microbial catabolism also occurs with A-type proanthocyanidins, since the additional ether linkage makes the interflavan linkage stiffer.^5,17^ Other factors influencing (poly)­phenol degradation at the colonic level are some physiological properties of the colonic environment, such as pH, bile salts, volume of liquid, and the composition and activity of the gut microbiota.^21–24^

Information about bacterial strains and enzymes involved in the bioconversion of flavan-3-ols is very limited. The production of phenyl-γ-valerolactones was positively correlated with members of clostridia and actinobacteria, as well as the genus .^25^ The relationship between (poly)­phenols, in particular flavan-3-ols, and gut microbiota is bidirectional: (poly)­phenols can modulate the activity and composition of the gut microbiota, and the gut microbiota can catabolize them, influencing their bioavailability and bioefficacy.^26^ In particular, (poly)­phenols have shown antimicrobial properties against potential pathogens (, , and certain Gram-negative ) and prebiotic effects by up-regulating some commensal and beneficial bacteria.^27–31^ In detail, (epi)­catechins have been shown to increase the growth of the group; proanthocyanidins have been associated with the growth of health-promoting bacteria, including , , and , and to a decrease of the Firmicutes/Bacteroides ratio; and red wine (poly)­phenols demonstrated the ability to enhance the abundance of beneficial bacteria (, , and ) at the expense of and .^32–34^ So, the protective effects deriving from the intake of flavan-3-ol-rich foods may depend not only on the production of phenolic catabolites but also on the microbiota-modulating effects that have been recently attributed to (poly)­phenols.

The present study aimed to optimize a validated protocol of *in vitro* colonic fermentation^17^ to mimic the colonic bioconversion of undigested compounds, particularly phytochemicals, and enhance the physiological relevance of the model. Optimization was carried out using three different flavan-3-ol substrates. This was achieved by extending the incubation time, monitoring the production of catabolites, and assessing the fermentation conditions, namely, pH and fecal microbiota composition.

## 2 Materials and Methods

### 2.1 Materials

All reagents and solvents were of analytical grade and were purchased from VWR International (Milan, Italy), unless otherwise indicated. Ultrapure water from a Milli-Q system (Millipore, Bedfort, MA, USA) was used throughout the experiment. Formic acid, bile salts, soluble starch, (+)-arabinogalactan, tryptone, yeast extract, xylan from birchwood, l-cysteine hydrochloride monohydrate, guar gum, inulin, Tween 80, buffered peptone water, Dulbecco’s phosphate buffer saline (PBS), casein sodium salt from bovine milk, pectin from citrus fruits, mucin from porcine stomach-type III, CaCl_2_, KCl, NaCl, NaHCO_3_, anhydrous K_2_HPO_4_, KH_2_PO_4_, MgSO_4_ monohydrate, FeSO_4_ heptahydrate, resazurin redox indicator, (−)-epicatechin (EC), phenylacetic acid, 4′-hydroxyphenylacetic acid, 3′-hydroxyphenylacetic acid, 2-(3′,4′-dihydroxyphenyl)­acetic acid, 3-phenylpropanoic acid, 3-(4′-hydroxyphenyl)­propanoic acid, 3-(3′-hydroxyphenyl)­propanoic acid, 3-(3′,4′-dihydroxyphenyl)­propanoic acid, benzoic acid, 4-hydroxybenzoic acid, 3-hydroxybenzoic acid, 3,4-dihydroxybenzoic acid, 1,3,5-trihydroxybenzene, 3,4-dihydroxybenzaldehyde, and 4-hydroxybenzaldehyde were purchased from Sigma-Aldrich (St Louis, MO, USA). Procyanidin dimers A2 and B2 were purchased from Extrasynthese (Genay Cedex, France). 5-(4′-Hydroxyphenyl)-valerolactone, 5-(3′-hydroxyphenyl)-valerolactone, and 5-(3′,4′-dihydroxyphenyl)-γ-valerolactone were synthesized in-house and kindly supplied by Prof. Curti using the synthetic strategy previously reported.^35^

### 2.2 *In Vitro* Fecal Fermentation

Three flavan-3-ols, including (−)-EC, procyanidin B2 (PC_B2), and procyanidin A2 (PC_A2), were used individually as substrates for microbial catabolism. *In vitro* colonic batch fermentation was performed following validated protocols, with some modifications.^17,36–39^ The growth medium (1 L) was prepared as previously reported,^37–40^ aliquoted and sterilized at 121 °C for 15 min in glass vessels (12 mL) before sample preparation. Fresh fecal samples were collected from three healthy donors with no previous intestinal diseases and who had not been treated with antibiotics for the previous 3 months. The Ethics Committee of Area Vasta Emilia Nord (AVEN) approved the study (protocol no. 796/2018/sper/unipr) and all the donors provided informed consent for the collection of fecal material. Donors followed a strict and controlled very low-(poly)­phenol diet for 2 days prior to fecal collection. Feces were immediately stored in anaerobic jars containing an anaerobic atmosphere generation bag to create an oxygen-free environment. An equal mass of feces from each donor was weighed, diluted with Dulbecco’s PBS (1%, w/v), and homogenized to obtain a 10% (w/w) fecal slurry to be used as the inoculum for fermentation. Feces were pooled to create a colonic model with a more representative microbiota composition and to avoid biases due to interindividual variability. Parent compounds were dissolved in an aqueous solution, and suspensions were left for 2 h at room temperature under constant magnetic stirring. Parent compounds were fermented at a final concentration of 75 μmol/L. This is in line with the concentration of flavan-3-ols found in the ileal fluid of ileostomy patients consuming dietary amounts of flavan-3-ols.^41,42^ In each fermentation batch, containing 45% of the growth medium and previously sterilized, an aliquot of 45% of fecal slurry and 10% of substrate suspension were added to reach a total fermentation volume of 4 mL. Vessels were rapidly sealed and flushed with N_2_ to create anaerobiosis before incubation at 37 °C at 200 strokes min^–1^ in a Dubnoff water bath (JULABO, Seelbach, Germany). Fermented samples were collected prior to starting the fecal fermentation (0 h) and after different incubation times: 5 h, 8 h, 24 h, 30 h, and 48 h. Blank samples, containing the culture medium and the fecal slurry, without substrate, as well as abiotic control samples, containing the culture medium and the substrate, without fecal slurry, were prepared and incubated as previously reported.^37–40^

The extension of the incubation time beyond 24 h was obtained with some additional steps after 24 and 30 h for samples incubated for 30 and 48 h, respectively. In particular, batches were degassed with a sterile syringe to reduce the pressure caused by the gases produced by microbiota fermentation. Fresh growth medium (1 mL) was added to the vessels prior to flushing N_2_ to ensure the maintenance of an anaerobic environment. To monitor the fermentation conditions, pH was measured at the end of every fermentation time, prior to stopping the microbiota activity, using a pH meter (Mettler-Toledo S.p.A., Milan, Italy). Finally, after the incubation period and pH measurements, microbial catabolism was stopped by adding 10% v/v of acetonitrile for (poly)­phenol catabolite analysis or adding 0.7 mL of an inactivating liquid acting as a DNA/RNA shield (Zymo Research, Irvine, CA, USA) to an aliquot (1 mL) of fermented sample for microbiota composition analysis. Samples were frozen (−80 °C) until extraction and analysis. All experiments were carried out in triplicate.

### 2.3 Fecal Metabolite Extraction

Native flavan-3-ols and phenolic catabolites generated during the *in vitro* fecal fermentation were extracted adopting a method previously reported,^17^ with slight modifications. An aliquot of 300 μL of each fermented sample was extracted by using 1200 μL of acidified methanol (0.1% v/v formic acid). The solution was vortexed for 2 min, sonicated for 10 min in an ultrasonic bath (Ultrasonic Cleaning Bath USC-THD, VWR, Radnor, PA, USA), vortexed for 2 min, and resonicated for 5 min. Samples were centrifuged (Centrisart A-14C Refrigerated Micro-Centrifuge and Rotor YCSR-A1C, Sartorius Lab Instruments GmbH and Co. KG, Goettingen, Germany) at 14 460 × *g* for 10 min, and the organic extract was transferred into a clean microfuge tube. After the first extraction, the residual pellet of the fermented samples was re-extracted following the same procedure, using 500 μL of the same solvent. Finally, the supernatants were pooled. An aliquot of 1 mL of supernatant was dried under vacuum by a centrifugal vacuum concentrator (SpeedVac Savant SPD121P, Thermo Fisher Scientific Inc., San Jose, CA, USA), and the dried pellet was resuspended with 200 μL of acidified methanol (0.1% v/v formic acid), vortexed for 2 min, sonicated for 10 min, and finally centrifuged for 5 min at 14 460*g* at 4 °C. The concentrated samples, as well as the remaining supernatant, were analyzed by ultrahigh performance liquid chromatography coupled with tandem mass spectrometry (uHPLC-MS/MS).

### 2.4 uHPLC-ESI-MS/MS Analysis

Organic fecal extracts were analyzed using a uHPLC DIONEX Ultimate 3000 fitted with a TSQ Vantage triple quadrupole mass spectrometer equipped with a heated-electrospray ionization source (H-ESIII; Thermo Fisher Scientific Inc.). Separation was carried out by means of a Kinetex Evo C18 column (100 mm × 2.1 mm; 2.6 μm particle size; Phenomenex, CA, USA), installed with a precolumn cartridge (Phenomenex). Chromatographic and ionization parameters were based on the method of Brindani et al.^43^ Briefly, the mobile phase, pumped at a flow rate of 0.4 mL/min, consisted of a mixture of acidified water (0.01% v/v formic acid) (solvent A) and acidified acetonitrile (0.01% v/v formic acid) (solvent B). Following 0.5 min of 5% solvent B in A, the proportion of B was increased linearly to 40% over a period of 7 min. Solvent B was increased again to 80% in 1 min and maintained for 2 min, and then the start conditions were re-established in 0.5 min and maintained for 3 min to re-equilibrate the column (total run: 14 min). The ESI source interface was set to a capillary temperature of 275 °C, and the source heater temperature was 250 °C. The sheath gas (N_2_), the auxiliary gas (N_2_), and the sweep gas flow rate were set at 40, 5, and 15 arbitrary units, respectively. The source voltage was 3 kV, the capillary voltage was −9 V, and the tube lens voltage was −53 V. Analyses were performed in negative ionization mode. A total of 40 compounds, including 3 parent compounds and 37 gut microbiota catabolites, were monitored through selective reaction monitoring mode (Table S1 in Supporting Information). Quantification was performed with calibration curves of standards, when available, or using the most structurally similar compound. Data processing was performed using Xcalibur software version 2.1 (Thermo Scientific Inc.).

### 2.5 Data and Statistical Analysis for Phenolic Compounds

Concentration values of microbial catabolites were expressed as mean values ± standard deviation (SD). In accordance with previous studies,^17,44^ molar mass recoveries for catabolites were calculated and expressed as percentages (%) based on the incubated concentration of the parent compound (75 μmol/L) to compare the different fermented substrates. One-way ANOVA with Tukey’s posthoc test was applied to detect differences in catabolite concentrations obtained from fecal fermentation of different substrates over the same incubation period (0, 5, 8, 24, 30, and 48 h) and to detect differences in catabolite production within the same substrate among different incubation periods (0 h, 5 h, 8 h, 24 h, 30 h, and 48 h). Differences were considered significant at *p*-value < 0.05. ANOVA was performed using IBM SPSS Statistics version 26 (IBM, Chicago, IL, USA).

Principal component analysis (PCA) was applied to explore differences in the behavior of the substrates in the *in vitro* colonic environment and in the appearance of catabolites over the fecal fermentation. PCA was carried out considering molar mass recoveries of all microbial catabolites before incubation (0 h) and during the different incubation times (5 h, 8 h, 24 h, 30 h, and 48 h). Data were pretreated with mean-centering plus Unit Variance scaling, also called autoscaling. Two principal components (PCs) were set. The parameter used to assess the quality of the model and subsequent data interpretability was R^2^X, namely, the model fit (or explained variation). PCA was performed using SIMCA 16.0.1 software (Sartorius Stedim Data Analytics, Umea, Sweden).

### 2.6 Fecal Bacterial DNA Extraction, 16S rRNA Gene Polymerase Chain Reaction Amplification, and DNA Sequencing

Fecal fermentations were collected and stored at −80 °C until microbiota analysis. Bacterial DNA was extracted using a QIAamp Fast DNA Stool Mini Kit (Qiagen, Germany) according to the manufacturer’s instructions. Partial 16S rRNA gene sequences were amplified from extracted DNA using primer pair Probio_Uni and/Probio_Rev, targeting the V3 region of the 16S rRNA gene sequence.^45^ Illumina adapter overhang nucleotide sequences were added to the partial 16S rRNA gene-specific amplicons, which were further processed by the 16S Metagenomic Sequencing Library Preparation Protocol (Part #15044223 Rev. B—Illumina). Amplifications were carried out using a Verity Thermocycler (Applied Biosystems). The integrity of the polymerase chain reaction (PCR) amplicons was analyzed by electrophoresis on a 2200 TapeStation Instrument (Agilent Technologies, USA). DNA products obtained following PCR-mediated amplification of the 16S rRNA gene sequences were purified by a magnetic purification step involving the Agencourt AMPure XP DNA purification beads (Beckman Coulter Genomics GmbH, Bernried, Germany) to remove primer dimers. A fluorimetric Qubit quantification system determined the DNA concentration of the amplified sequence library (Life Technologies, USA). Amplicons were diluted to a concentration of 4 nM, and 5 μL quantities of each diluted DNA amplicon sample were mixed to prepare the pooled final Library. Sequencing was performed using an Illumina MiSeq sequencer with MiSeq Reagent Kit v3 chemicals.

### 2.7 Bioinformatics and Statistical Analysis for Microbiota Data

The fastq files obtained from sequencing were processed using QIIME2,^46,47^ as previously described.^45^ Paired-end reads were merged, and sequences that passed quality control, with a length between 140 and 400 bp, a mean sequence quality score >25, and with truncation of a sequence at the first base if a low-quality rolling 10 bp window, were found. Sequences with mismatched forward and/or reverse primers were removed.

In order to calculate downstream diversity measures (alpha and beta diversity indices), 16S rRNA amplicon sequence variants (ASVs) were defined at 100% sequence homology using DADA2.^48^ ASVs not encompassing at least 2 sequences of the same sample were removed. Notably, this approach allows highly distinctive taxonomic classification at single nucleotide accuracy.^48^ All reads were classified to the lowest possible taxonomic rank using QIIME2 ^46,47^ and a reference data set from the SILVA database version 123.^49^ Biodiversity within a given sample (alpha-diversity) was calculated with the observed ASVs index for ten subsamplings of sequenced read pools and represented by rarefaction curves. Similarities between samples (beta-diversity) were calculated by Bary–Curtis dissimilarity.^50^ Similarity scores were calculated as values between 0 and 1. Principal coordinates analysis (PCoA) representations of beta-diversity were performed using QIIME2.^46,47^ PERMANOVA analyses were performed on QIIME2 to estimate possible significant differences between the sample groups. Moreover, the Bray–Curtis dissimilarity matrix was used to evaluate the distance between samples at different time points. Furthermore, the correlation analysis between the available bacterial taxa and catabolites was performed through Spearman’s rank correlation coefficient using the “rcorr” function (from Hmisc_4.6–0, https://CRAN.R-project.org/package=Hmisc). The FDR correction based on the Benjamini and Hochberg correction^51^ and calculated using RStudio through the “p.adjust” function (from the base package stats) was applied to statistically significant results.

### 2.8 Data Deposition

The 16S rRNA microbial profiling data sets achieved in this study were deposited in SRA under accession no. PRJNA1082543.

## 3 Results and Discussion

### 3.1 Identification and Quantification of Microbial Catabolites

EC, PC_B2, and PC_A2 were incubated for 5, 8, 24, 30, and 48 h at a concentration of 75 μmol/L. After 5 h of incubation, the decrease of the parent compound was 72% for PC_A2 and 100% for PC_B2 and EC, in line with data previously obtained.^17^ This confirmed that the degradation of PC_B2 and EC is more rapid than the catabolism of PC_A2 due to differences in the structure and chemical linkages.

A total of 37 microbial catabolites were monitored^7,52–54^ based on the available bioconversion pattern for monomeric and dimeric flavan-3-ols and on previous *in vitro* fermentation studies.^17,18,38,39,44,55–64^ A total of 14 catabolites were identified and quantified in samples after fecal incubation. In blank samples, consisting of growth medium and fecal material without flavan-3-ol substrates, small amounts of phenolic catabolites were detected, in particular 3-(3′-hydroxyphenyl)­propanoic acid, 4-hydroxybenzoic acid, and 4-hydroxybenzaldehyde. These quantities were subtracted from the amounts found in the substrate-containing fermented samples at each incubation time. In abiotic control samples, consisting of substrates and growth medium without the fecal material, no more than negligible amounts of catabolites were detected. These data indicated that the very low-(poly)­phenol diet produced almost blank feces and that the incubation process did not influence the production of the quantified catabolites.

Quantified catabolites, mainly produced within the first incubation period, presented different chemical structures, namely, monomers (*n* = 1), originating from the fission of the interflavan bond of dimers; fission dimers (*n* = 2), presenting a C-ring opening of one monomeric unit; and diphenylpropan-2-ols (*n* = 2), derived from the C-ring fission of monomers. After native structure modification, flavan-3-ols were further catabolized by colonic microbiota into lower molecular weight compounds, including phenyl-γ-valerolactones (*n* = 2), derived from the A-ring fission of diphenylpropan-2-ols or possibly from the degradation of the lower unit of dimers; phenylvaleric acids (n = 3), resulting from the hydrolysis of the lactone ring of phenyl-γ-valerolactones; phenylpropanoic acids (*n* = 1), originating from the β-oxidation of phenylvaleric acids; phenylacetic acids (*n* = 1), arising from a further α-oxidation of phenylpropanoic acids or from the degradation of the upper unit of dimers; benzoic acids (*n* = 1), originating from the β-oxidation of phenylpropanoic acids or the α-oxidation of phenylacetic acids; and benzaldehydes (*n* = 1), obtained after the reduction of the carboxyl group of benzoic acids.

Comparing the substrates, PC_B2 was characterized by the richest catabolic profile from a qualitative point of view, resulting in the production of 13 metabolites, followed by EC, accounting for 10 metabolites. From a quantitative point of view, EC was the substrate leading to the richest production, being the most catabolized parent compound, followed by PC_B2. PC_A2 was characterized by the poorest profile both from a qualitative and quantitative point of view, displaying only 5 catabolites and having the lowest concentration of quantified compounds (Table S2 in Supporting Information). Analyzing the kinetics, the peak of catabolites was obtained after 8 and 24 h of incubation for EC and procyanidins, respectively, reflecting their catabolic rate and the different hindrance to degradation between monomers and dimers, in accordance with existing evidence.^17–20^ As previously shown,^17^ phenyl-γ-valerolactones were the main class of catabolites for EC and PC_B2, while fission dimer was the characterizing catabolite for PC_A2. Production of phenyl-γ-valerolactones reached its maximum value at 8 h for EC, 24 h for PC_B2, and 48 h for PC_A2 (Figure 1A), suggesting that PC_B2 and PC_A2 also have different catabolic rates. The extension of the fermentation time until 48 h and the addition of time 8 allowed us to have a more comprehensive view of flavan-3-ol catabolism from a kinetic point of view, suggesting the need to reach longer in vitro fermentation time to allow a proper catabolism of some undigested (poly)­phenols.

**Figure 1:**
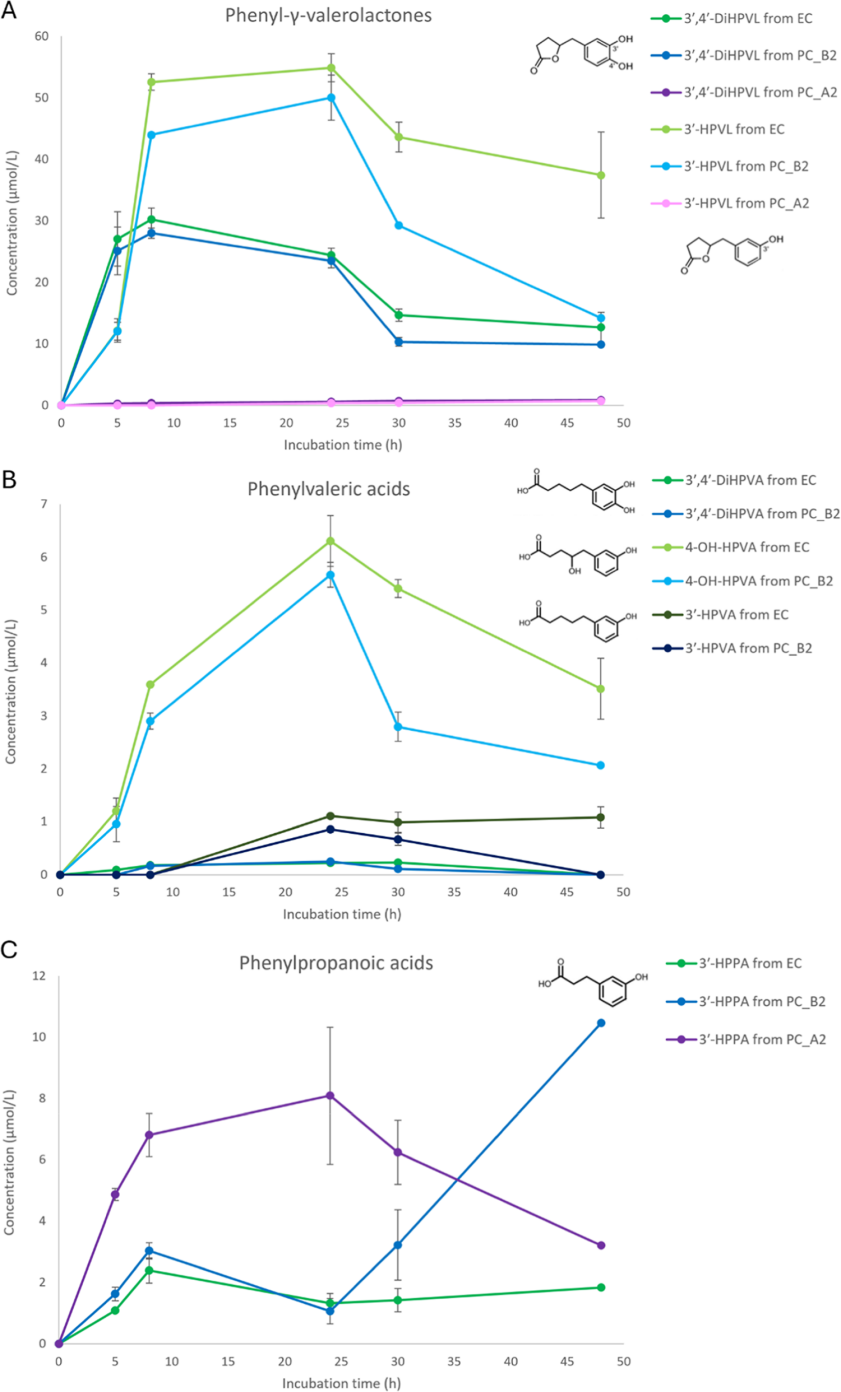
Kinetics of the main microbial catabolites obtained after 5, 8, 24, 30, and 48 h of fecal fermentation incubating (−)-epicatechin (EC), procyanidin dimer B2 (PC_B2), and procyanidin dimer A2 (PC_A2). The catabolites belong to the classes of (A) phenyl-γ-valerolactones, (B) phenylvaleric acids, and (C) phenylpropanoic acids. Concentration data (μmol/L) are expressed as the mean ± SD (*n* = 3). 3′,4′-diHPVL: 5-(3′,4′-dihydroxyphenyl)-γ-valerolactone; 3′-HPVL: 5-(3′-hydroxyphenyl)-γ-valerolactone; 3′,4′-diHPVA: 5-(3′,4′-dihydroxyphenyl)­valeric acid; 4-OH-HPVA: 4-hydroxy-5-(hydroxyphenyl)­valeric acid; 3′-HPVA: 5-(3′-hydroxyphenyl)­valeric acid; and 3′-HPPA: 3-(3′-hydroxyphenyl)­propanoic acid. Statistics is reported in Supporting Information file (Table S2).

### 3.2 Catabolism and Production of Microbial Catabolites

Considering the concentration values for each microbial catabolite, comparisons were performed among different substrates within the same fermentation time and among different incubation periods applied to the same substrate (Table S2). EC was produced in small amounts after 5 h incubation of PC_B2, indicating that the cleavage of the interflavan bond is a minor pathway (<10%) for B-type procyanidins, as observed in other studies.^17,18^ Fission catabolites, derived from the cleavage of one C-ring of procyanidins, were produced from both dimers, but in different quantities: 1-fission dimer A2 was the main catabolite of PC_A2, reaching a molar mass recovery of 53% at 24 h, while for PC_B2, the fission dimer was a minor catabolite produced until 8 h, with a second peak at 48 h. A PC_B2 fission catabolite presenting two cleavages was not identified. Diphenylpropan-2-ols in their mono- and dihydroxylated forms on the B-ring appeared only after 5 and 8 h incubation of EC and PC_B2, as expected.^17,18,58^ As noticed for 1-fission dimer B2, a second peak of 1-(hydroxyphenyl)-3-(2″,4″,6″-trihydroxyphenyl)-propan-2-ol appeared at 48 h, suggesting that upstream catabolites, normally produced in the first incubation hours, could also be generated after 30 h for B-type procyanidins. This could be due to a recovery of microbial catabolism after the microbiota-driven release of procyanidins that might interact with components of the fermentation medium, such as proteins. The presence of proteins in the donor feces was expected because volunteers followed a very low-(poly)­phenol diet, avoiding plant-based foods rich in fibers while increasing the intake of animal foods, relevant sources of proteins. It is well-known that tannins, including proanthocyanidins, interact with proteins in the gut lumen, limiting the access to the C-ring by the colonic microbiota and slowing down their catabolism.^65^

As regards to low molecular weight molecules, phenyl-γ-valerolactones were found to be the most important catabolites for EC and PC_B2 and minor catabolites for PC_A2 (Figure 1A), confirming that A-type PCs are more resistant to microbial catabolism, in particular for the A-ring fission step.^5,17,44^ The presence of an additional ether bond in A-type procyanidins makes their structure stiffer and less subject to degradation. 5-(3′-Hydroxyphenyl)-γ-valerolactone was the main catabolite obtained from EC and PC_B2 incubation, and it reached its maximum concentration at 24 h for EC and PC_B2 and at 48 h for PC_A2 (Figure 1A). This indicated that the substrates have a different degradation rate due to differences in the steric hindrance and structure stiffness.^17,18,20,44^ Similarly to 5-(3′-hydroxyphenyl)-γ-valerolactone, 5-(3′,4′-dihydroxyphenyl)-γ-valerolactone showed different kinetics according to the substrate: it reached its production peak after 8 h incubation for EC and PC_B2, and at 48 h for PC_A2 (Figure 1A). The fermentation until 48 h allowed the detection of 5-(3′-hydroxyphenyl)-γ-valerolactone as a catabolite of PC_A2, in addition to 5-(3′,4′-dihydroxyphenyl)-γ-valerolactone, previously reported.^5,17,44^ These results indicated that the catabolism of PC_A2 increased after 24 h, giving importance to the extension of the incubation time until 48 h. Moreover, the maximum yield of 5-(3′,4′-dihydroxyphenyl)-γ-valerolactone detected at 8 h suggested that adding this fermentation time could be crucial in the study of the degradation pathways of monomers. The delayed peak of 5-(3′-hydroxyphenyl)-γ-valerolactone compared to 5-(3′,4′-dihydroxyphenyl)-γ-valerolactone has been observed also in other studies^17,44,66^ and reflects the timing of the catabolic pathway, since 3′-monohydroxylated catabolites derive from 4′-dehydroxylation of 3′,4′-dihydroxylated precursors.

Three different phenylvaleric acids were quantified after EC and PC_B2 fecal incubation but not for PC_A2, and their kinetics and relative abundance were in accordance with the data obtained by Di Pede et al.^17^ These phenylvaleric acids were also detected in other studies investigating the *in vitro* microbial conversion of flavan-3-ol monomers and B-type dimers.^18,44,56,67^ All phenylvaleric acids reached their maximum concentrations at 24 h for both substrates (Figure 1B). 4-Hydroxy-5-(hydroxyphenyl)­valeric acid was the main phenylvaleric acid, followed by 5-(3′-hydroxyphenyl)­valeric acid, while 5-(3′,4′-dihydroxyphenyl)­valeric acid was the minor one (Figure 1B). This confirms that 4-hydroxy-phenylvaleric acids, generated by the hydrolysis of phenyl-γ-valerolactones, are more abundant than their corresponding 4-dehydroxylated forms.^5^ Nevertheless, the abundance of 3′-hydroxyl derivatives of phenyl-γ-valerolactones and phenylpropanoic acids suggested that the 4′-hydroxyl group is preferentially removed than the 3′-hydroxyl group from dihydroxylated precursors, as observed in other studies.^18,44,56,66,67^

Considering small phenolic acids, 3-(3′-hydroxyphenyl)­propanoic acid was quantified in all samples. This compound showed a biphasic behavior for EC and PC_B2, reaching its peak both at 8 h and at 48 h (Figure 1C), suggesting that it could derive from two different pathways, namely, the β-oxidation of 3′-monohydroxylated phenylvaleric acid and the 4′-dehydroxylation of 3′,4′-dihydroxylated phenylpropanoic acids, the latter not quantified here but reported in other studies.^56,67^ The monitoring of five different incubation times allowed to understand properly the kinetics of 3-(3′-hydroxyphenyl)­propanoic acid. 3-(3′-Hydroxyphenyl)­propanoic acid was identified as a characterizing metabolite for PC_A2, reaching a recovery of 11% at 24 h, in accordance with other studies.^17,44^

The extension of the incubation time until 48 h and the addition of time 8 allowed us to quantify other small phenolic acids, including 2-(3′,4′-dihydroxyphenyl)­acetic acid, 4-hydroxybenzoic acid, and 4-hydroxybenzaldehyde, which were not previously reported.^17^ In line with existing evidence,^44,56,66^ 2-(3′,4′-dihydroxyphenyl)­acetic acid resulted to be a catabolite specific for PC_B2, being quantified after 5 and 8 h of fermentation, and reaching its maximum concentration at 5 h. These data suggest that this compound arises from the quick degradation of the upper units of B-type dimers. Small quantities of 4-hydroxybenzoic acid were found at 48 h for all substrates and at 30 h for EC, suggesting a delayed production, as expected for final catabolites.^7^ A study by Sánchez-Patán et al.^66^ confirmed the production of 4-hydroxybenzoic acid as end-product of grape seed flavan-3-ol fermentation. 4-Hydroxybenzoic acid and its dehydroxylated benzoic acid derivative are precursors of 4-hydroxyhippuric acid and hippuric acid, respectively, which are important *in vivo* glycine-conjugated metabolites of flavan-3-ols.^7^ Finally, 4-hydroxybenzaldehyde was quantified for EC and PC_B2 after different incubation times: for EC, it appeared after 30 and 48 h incubation, and for PC_B2, it was generated after 8 and 24 h, suggesting different kinetics for monomers and proanthocyanidins. This is the first time that 4-hydroxybenzaldehyde has been reported in an *in vitro* fermentation study using flavan-3-ols.

### 3.3 Assessment of the Differences in Microbial Catabolism of the Incubated Flavan-3-ols

Unsupervised multivariate analysis (PCA) was applied to explore differences in the behavior of the substrates in the *in vitro* colonic environment and in the time of appearance of catabolites over the fecal incubation period (Figure 2). Two PCs explained up to 62.9% of the total variability, with PC1 and PC2 explaining 36.4% and 26.5%, respectively. PC1 was positively loaded mainly by metabolites characterizing PC_A2, namely, 1-fission dimer A2 and 3-(3′-hydroxyphenyl)­propanoic acid, and was negatively loaded by those metabolites abundant after EC and PC_B2 incubation, including phenyl-γ-valerolactones, phenylvaleric acids, 4-hydroxybenzoic acid, and 4-hydroxybenzaldehyde. PC2 was positively loaded by early produced catabolites characterizing EC and PC_B2 in the first incubation times (5 and 8 h), including EC, 1-fission dimer B2, diphenylpropan-2-ols, 5-(3′,4′-dihydroxyphenyl)-γ-valerolactone, and 2-(3′,4′-dihydroxyphenyl)­acetic acid.

**Figure 2:**
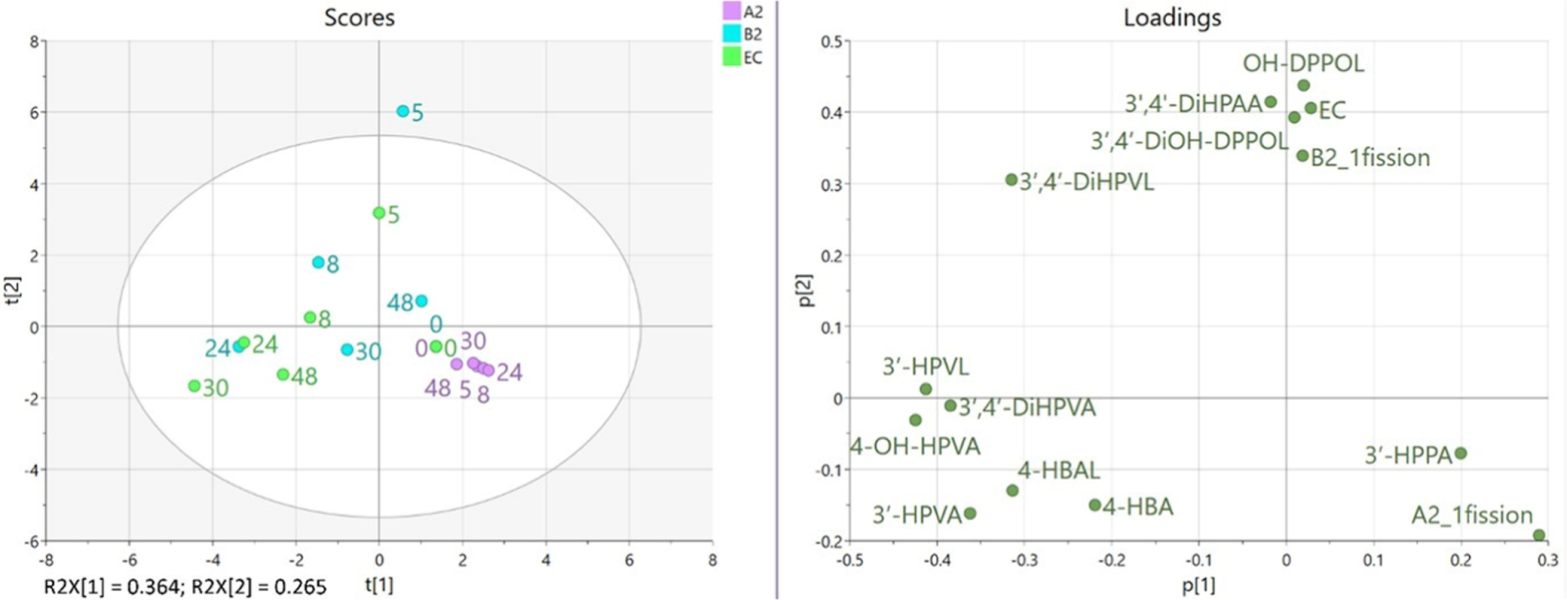
Score and loading plots resulting after applying PCA on autoscaled data. Observations represent molar mass recoveries for microbial metabolites at each fermentation time (T0, T5, T8, T24, T30, and T48) for the substrates procyanidin dimer A2 (PC_A2), procyanidin dimer B2 (PC_B2), and (−)-EC. A2_1fission: 1-fission dimer A2; B2_1fission: 1-fission dimer B2; 3′,4′-diOH-DPPOL: 1-(3′,4′-dihydroxyphenyl)-3-(2″,4″,6″-trihydroxyphenyl)-propan-2-ol; OH-DPPOL: 1-(hydroxyphenyl)-3-(2″,4″,6″-trihydroxyphenyl)-propan-2-ol; 3′,4′-diHPVL: 5-(3′,4′-dihydroxyphenyl)-γ-valerolactone; 3′-HPVL: 5-(3′-hydroxyphenyl)-γ-valerolactone; 3′,4′-diHPVA: 5-(3′,4′-dihydroxyphenyl)­valeric acid; 4-OH-HPVA: 4-hydroxy-5-(hydroxyphenyl)­valeric acid; 3′-HPVA: 5-(3′-hydroxyphenyl)­valeric acid; 3′-HPPA: 3-(3′-hydroxyphenyl)­propanoic acid; 3′,4′-diHPAA: 3′,4′-dihydroxyphenylacetic acid; 4-HBA: 4-hydroxybenzoic acid; and 4-HBAL: 4-hydroxybenzaldehyde.

Considering the score plot (Figure 2), samples were discriminated by PC1 according to the substrate and by PC2 according to the incubation time. PC_A2 observations at all incubation times lay in the same quadrant (positive values for PC1 and negative values for PC2) and were clustered together. This confirmed that PC_A2 was subjected to a different catabolism than EC and PC_B2, suggesting also that the catabolic profile of this substrate did not change significantly during the 48 h incubation period. On the contrary, EC and PC_B2 observations clustered together according to the incubation time: for early time points (5 and 8 h), observations lay in the upper quadrants (positive PC2), while for later times, including 24, 30, and 48 h, observations lay in the bottom left quadrants (negative PC1 and PC2), with the exception of time 48 for PC_B2, located in the upper right quadrant (positive PC1 and PC2). This indicates that EC and PC_B2 were catabolized in a similar way and that the catabolic profile was driven mainly by the incubation time. The only difference between the incubated substrates was the kinetics: PC_B2 showed a slower catabolism, resulting in the shifting of times 5 and 8 in the upper part of the quadrants compared to EC, evaluated within the same incubation period. After 5 h of microbiota catabolism, the profile of EC and PC_B2 was characterized by diphenylpropan-2-ols, 5-(3′,4′-dihydroxyphenyl)-γ-valerolactone, and catabolites specific for PC_B2, namely, EC, 1-fission dimer B2, and 2-(3′,4′-dihydroxyphenyl)­acetic acid. 5-(3′,4′-Dihydroxyphenyl)-γ-valerolactone characterized the catabolite production profile of both substrates obtained after 8 h incubation. On the contrary, in late incubation times (24, 30, and 48 h), the most relevant catabolites were low molecular weight compounds, including 5-(3′-hydroxyphenyl)-γ-valerolactone, phenylvaleric acids, 3-(3′-hydroxyphenyl)­propanoic acid, 4-hydroxybenzoic acid, and 4-hydroxybenzaldehyde.

### 3.4 Assessment of the Stability of the Fermentation Environment: Measurement of pH

The fermentation conditions were monitored through pH measurements and fecal microbiota composition analysis in the different incubation times. It is known that the colonic pH can deeply influence the composition and metabolic activity of the gut microbiota, affecting the activity of enzymes involved in the fermentation of prebiotics.^68^ In this study, the microbial metabolic activity showed a similar effect on the acid–base balance for each substrate: pH ranged from 7 to 8 at 0 h and decreased after 5 h of fermentation, reaching a value between 5 and 6, remaining stable until 48 h, with only slight fluctuations (Figure S1 in Supporting Information).

The pH decrease was probably due to the formation of microbial catabolites, such as phenolic acids, lactic acid, and short-chain fatty acids (SCFAs), namely, acetic, propionic, and butyric acid.^69–71^ Low molecular weight phenolic acids were mainly produced through fermentation of the native incubated (poly)­phenol substrates, although a minor part can arise from the microbial catabolism of aromatic amino acids present in the growth medium,^72^ as well as possibly contained in donor feces because of the very low-(poly)­phenol diet.^73,74^ SCFAs would arise from the breakdown of fermentable dietary fibers added to the growth medium, such as arabinogalactan, xylan, guar gum, inulin, and pectin,^75^ and, to a lesser extent, from the catabolism of amino acids derived from proteins contained in donor feces and medium proteins, including tryptone, yeast extract, peptone, casein, and mucin.^72^ It is known that pectin metabolism produces acetate, while inulin increases the molar proportion of butyrate.^70^ The saccharolytic activity of the fecal microbiota reproduced the colonic environment, in which fibers are fermented, maintaining the pH around 5.5.^21,69,70^ This confirms that the *in vitro* fermentation environment mimics colonic features and is optimal for microbial activity. Moreover, the decrease of pH also in blank controls suggested that fibers and amino acids present in the medium and feces may be the main sources of organic acids responsible for environment acidification, rather than (poly)­phenols.

### 3.5 Assessment of the Stability of the Fermentation Environment: Analysis of the Microbiota Composition

Catabolism of (poly)­phenols and dietary fibers can also affect the composition and activity of the gut microbiota.^21,76^ Moreover, a specific microbiota composition is crucial to reach good fermentation activity. In this context, the possible impact that the three different flavan-3-ols may have on the fecal microbiota composition was investigated. For this purpose, the collected fecal sample (FT) as well as the fermented samples at various time points were subjected to a 16S-rRNA microbial profiling approach (Table S3 in Supporting Information). This methodological approach was suggested in a recent study aiming to establish an *in vitro* batch fermentation protocol for studying the contribution of food to gut microbiota composition and functionality.^77^

It is well-known that the healthy gut ecosystem is characterized by the presence of anaerobic bacteria belonging to the Bacteroidetes and Firmicutes phyla, contributing to more than 90% of the total bacterial species. Other minor phyla are Proteobacteria, Actinobacteria, and Verrucomicrobia.^21^ These taxa have been identified by analyzing the microbial composition of fermented samples and controls in terms of phyla, families, and genera. As expected, the main phyla were Bacteroidetes and Firmicutes, and the Firmicutes/Bacteroidetes ratio ranged from 0.4 to 1.6, maintaining itself in a physiological concentration range (0.1–10) (Figure 3A).^78^ Proteobacteria increased at 8 h in both fermented samples and in controls, and this increase was mainly due to the genera and , belonging to the Enterobacteriaceae family. This indicates that some facultative anaerobes () and microaerophiles () commonly present in the intestine were able to grow, probably during the first early step of the fermentation, prior to recreating the anaerobic conditions. However, their abundance remained low, ranging from 2 to 5%, and did not compromise the microbial balance. Only a sample, namely, PC_B2 fermented for 48 h, showed a percentage of Proteobacteria of 10%, mainly determined by a spread of Enterobacteriaceae. Moreover, this sample, along with PC_B2 incubated for 30 h, showed an inversion of the Firmicutes/Bacteroidetes ratio, with Firmicutes more abundant than Bacteroidetes. This was mainly due to an increase of Clostridiaceae 1 and Acidaminococcaceae and a decrease of Prevotellaceae, which are known to have beneficial effects and are associated with SCFA production.^78^

**Figure 3:**
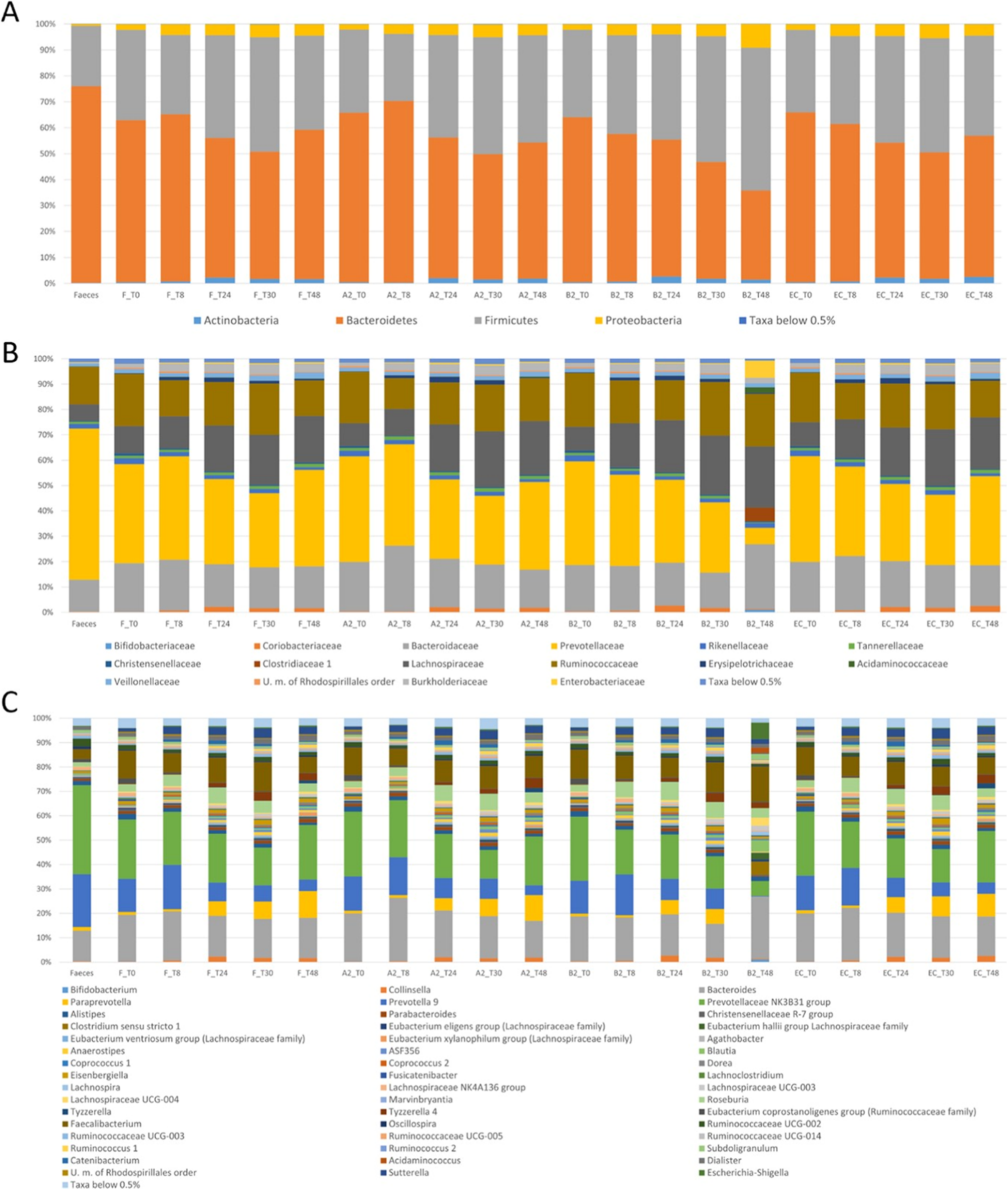
Composition of the fecal microbiota in a frozen fecal sample (feces) and at each incubation time (T0, T8, T24, T30, and T48) in blank controls (F) containing feces and growth medium, and in fermented samples for the substrates procyanidin dimer A2 (A2), procyanidin dimer B2 (B2), and (−)-EC. The composition is analyzed in terms of taxonomical classification in (A) phyla, (B) families, and (C) genera.

As happens for Proteobacteria, Actinobacteria increased at 8 h due to a spread of , except for PC_B2 fermented for 48 h, which was characterized by an increase of the genus. These data are interesting since three species belonging to the family, including JCM 16059^T^, JCM 14793^T^, and three strains of ( CAT-1, Rk3, and SDG-1) are able to cleave the C-ring of monomeric flavan-3-ols, forming diphenylpropan-2-ols. CAT-1 and JCM 14793^T^ can also dehydroxylate flavan-3-ols, phenylvaleric acids, and phenyl-γ-valerolactones at the B-ring, usually in 4′ position. Two strains of ( DSM 6740 and Ak2), a member of the family, can further degrade the C-ring fission products, catalyzing the A-ring cleavage and the hydrolysis of the lactone ring, giving origin to phenyl-γ-valerolactones and phenylvaleric acids.^5,79^ The Ruminococcaceae family was one of the main families and remained stable during the incubation. Other important families were Bacteroidaceae, Prevotellaceae, and Lachnospiraceae, the latter increasing after 24 h fermentation (Figure 3B). The principal genera were , 9, NK3B31 group, , and UCG-002 (Figure 3C). Interestingly, while 9 and NK3B31 decreased after 24 h fermentation, increased, leaving the abundance of the Prevotellaceae family almost unchanged. Moreover, the level of decreased after 8 h of *in vitro* fermentation.

### 3.6 Analysis of Microbial Diversity and Correlation between Microbial Composition and Catabolite Production

As expected, the metagenomic analysis showed that the alpha-diversity of the four incubation conditions (blank control, incubation of PC_A2, incubation of PC_B2, and incubation of EC) was similar at 0 h, thus suggesting that the inoculum was comparable across conditions. Moreover, blank control and incubation of both procyanidins followed a similar alpha-diversity trend, with the highest alpha-diversity values recorded after 30 h of fermentation and a parallel pronounced decrease at 48 h (Figure 4A). In contrast, when the growth medium was supplemented with EC, the highest biodiversity was reached after 24 h of fermentation and remained stable up to 48 h even if a slight alpha-diversity decline was observed in the intermediate time point (30 h; Figure 4A), suggesting a distinct microbial resilience probably related to the different supplemented substrate. However, despite these differences in the alpha-diversity trend, an overall increment of the alpha-diversity after 24 h was observed, suggesting that extending the fermentation time beyond 24 h may favor the proliferation of underrepresented bacterial genera. This proliferation could be related to the fermentation of the (poly)­phenolic substrate, thus explaining why the highest alpha diversity was reached at 24 h for EC, which was the substrate catabolized more rapidly, having its maximum recovery of catabolites at 8 h, and at 30 h for dimers, which were catabolized more slowly, showing a maximum recovery at 24 h.

**Figure 4:**
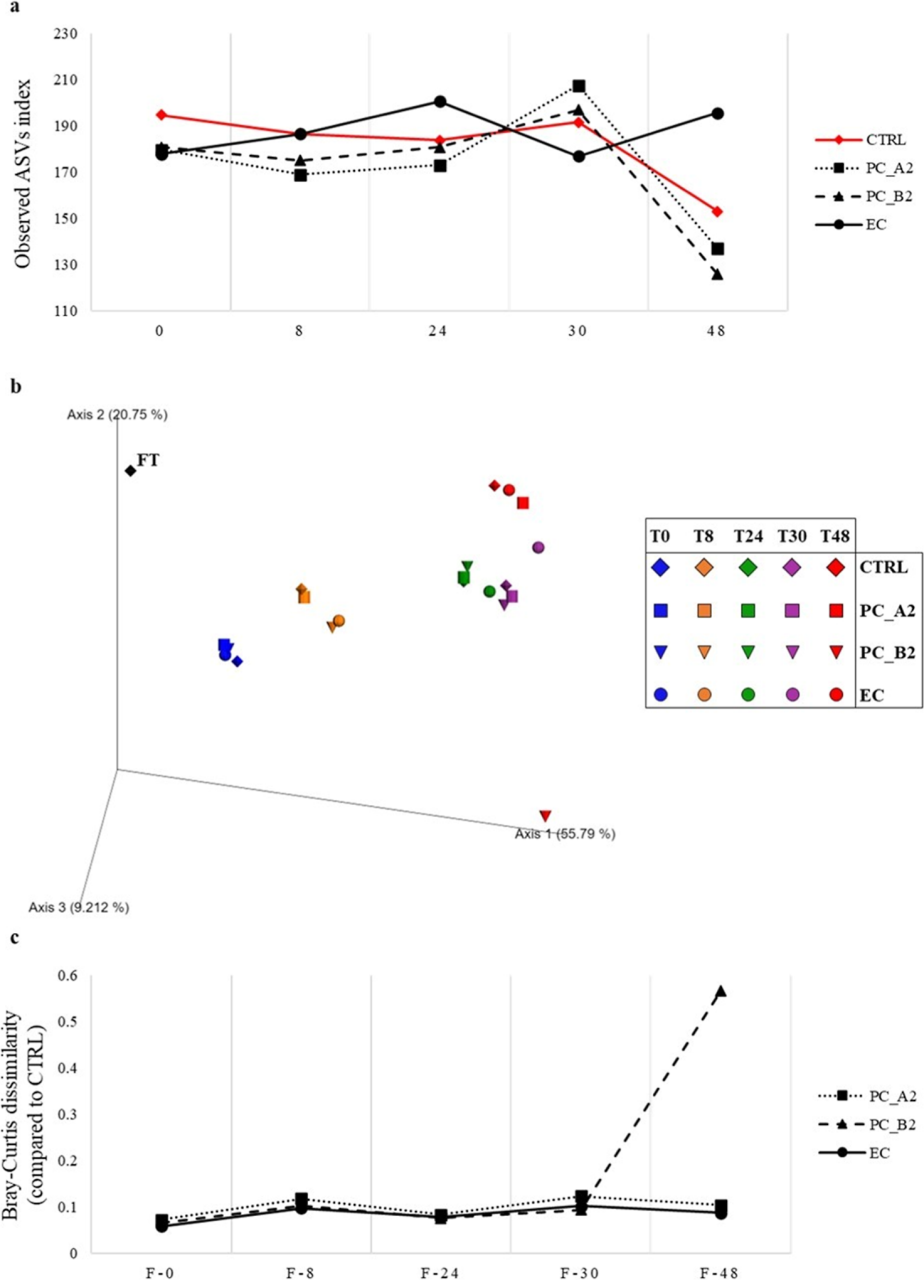
Evaluation of microbial biodiversity. Panel (A) displays the curves representing the alpha-diversity of each typology of fermentation over time. FT represents the alpha-diversity of the frozen fresh fecal sample. Panel (B) shows the principal coordinate analysis (PCoA) representing the distribution of the samples analyzed according to the microbiota composition. The samples included a frozen fresh fecal sample (FT-black color), blank controls (CTRL), and fermented samples at each fermentation time for the substrates procyanidin dimer A2 (PC_A2), procyanidin dimer B2 (PC_B2), and (−)-EC. Panel (C) reports the curves representing the statistical distances between CTRL and the other three fermentative conditions compared within the Bray–Curtis matrix for each time point.

Additionally, beta-diversity analysis based on Bray–Curtis dissimilarity and represented through a PCoA revealed sample clustering based on the incubation duration (PERMANOVA; *p*-value < 0.05) (Figure 4B), indicating a significant impact of the fermentation time on microbial composition. To further explore differences in bacterial composition within each cluster, statistical distances between the blank control and the three incubated substrates were compared within the Bray–Curtis matrix for each time point. The analysis revealed a slight deviation from the blank control in most samples, except at time 48, where the PC_B2 condition appeared to have a markedly different composition compared to the blank control (Figure 4C). Therefore, the microbiota composition in terms of genera resulted similarly in all samples except for PC_B2 at 48 h (Figures 4B,C, and S1). Since the performed in vitro experiment is of the preliminary nature, this aspect needs to be further investigated in future studies. Thus, the factor that mainly contributed to sample variability seemed to be the incubation time, suggesting that the presence and type of substrate did not significantly impact the relative abundance of microbial genera.

Furthermore, a preliminary correlation analysis based on Spearman’s rank correlation coefficient was performed to identify possible associations between bacterial taxonomy and catabolites. In detail, the analysis highlighted eight catabolites positively correlated with several bacterial genera belonging mainly to the Clostridiaceae and Lachnospiraceae families, while only catabolite 3-(3′-hydroxyphenyl)­propanoic acid revealed a negative correlation with , R-7 group, and NK3B31 group (Table 1). In particular, several specific flavan-3-ol metabolites, including 5-(3′,4′-dihydroxyphenyl)-γ-valerolactone, 5-(3′-hydroxyphenyl)-γ-valerolactone, and 4-hydroxy-5-(hydroxyphenyl)­valeric acid, positively correlated with sensu stricto and . The former correlated also with early catabolites, including 1-fission dimer B2 and 1-(hydroxyphenyl)-3-(2″,4″,6″-trihydroxyphenyl)-propan-2-ol. These results are interesting since sensu stricto 1 includes strictly anaerobic, fermenting bacteria of the human gut, which can metabolize various compounds, including carbohydrates, amino acids, alcohols, and purines, producing mainly butyric acid (a genus-specific product of fermentation) and varying amounts of other fermentation products (acetic acid, lactic acid, ethanol, propanol, and butanol).^80^ Moreover, the genus includes the species , formerly classified under the genus (as ), which has been shown to increase during the fermentation of (+)-catechin and (−)-EC.^33^ This preliminary correlation analysis highlighted a putative relationship between specific taxa and catabolites, possibly suggesting a link between the composition of the microbiota and catabolites produced during fermentation. While the microbiota analysis employed is appropriate for detecting taxonomic shifts, it does not allow for direct associations between specific microbial taxa and distinct catabolic reactions, such as C-ring cleavage or dihydroxylation, nor does it provide mechanistic insights. The correlation analysis presented in Table 1 is indicative of potential associations; however, a larger sample size is necessary to enhance the robustness and reliability of these findings. Additionally, an integrated approach incorporating shotgun metagenomics could offer deeper insight into microbial functionality and its relationship with polyphenol catabolite profiles.

**Table 1:** Correlation Analysis between the Bacterial Taxa Identified and Catabolites^a^

catabolites	taxa	Spearman’s rank correlation coefficient	*p*-value
5-(3′-hydroxyphenyl)valeric acid (3′-HPVA)		0.605516	0.033
		0.576923	0.048
3-(3′-hydroxyphenyl)propanoic acid (3′-HPPA)		0.583458647	0.044
	R-7 group	–0.661654135	0.014
		–0.686253571	0.010
	NK3B31 group	–0.735338346	0.004
4-hydroxybenzaldehyde (4-HBAL)		0.735772	0.004
		0.607627	0.032
		0.601686	0.034
	2	0.583865	0.044
		0.58047	0.046
fission dimer B2 (1-fission B2)	sensu stricto 1	0.649097	0.017
1-(hydroxyphenyl)-3-(2″,4″,6″-trihydroxyphenyl)-propan-2-ol (OH-DPPOL)	sensu stricto 1	0.649097	0.017
5-(3′,4′-dihydroxyphenyl)-γ-valerolactone (3′,4′-DiHPVL)	sensu stricto 1	0.608869	0.031
		0.695246	0.008
5-(3′-hydroxyphenyl)-γ-valerolactone (3′-HPVL)		0.679498903	0.011
	sensu stricto 1	0.58883346	0.041
	group (Lachnospiraceae family)	0.585533934	0.043
		0.580786361	0.046
4-hydroxy-5-(hydroxyphenyl)valeric acid (4-OH-HPVA)	sensu stricto 1	0.652894	0.016
		0.596595	0.037

a Only the statistical significance correlations were reported (*p* value < 0.05). The nomenclature of catabolites was based on Kay et al.^87^

The optimization of the *in vitro* colonic fermentation protocol confirmed the effect of flavan-3-ol structure on microbial catabolism from a quantitative, qualitative, and kinetic point of view. Flavan-3-ol polymerization and the inclusion of an ether linkage in A-type procyanidins, which increased the steric hindrance and stiffness of the molecule, limited the access to the C-ring and the A-ring fission obtained through gut microbiota activity, with a reduction of bioaccessibility and a delay in native structure catabolism. The extension of incubation time until 48 h and the monitoring of 5 different incubation times resulted in a more comprehensive view of flavan-3-ol kinetics and allowed quantification of small phenolic acids, including a catabolite specific of PC_B2 and two other small catabolites, one of which was not previously reported after flavan-3-ol *in vitro* fermentation. In this way, the extension of the incubation time until 48 h has demonstrated to maintain a physiological relevance, as expected based on the average colonic transit time in healthy people (48 h)^81^ and on existing evidence about excretion of (poly)­phenol metabolites in the urine collected between 24 and 48 h after (poly)­phenol intake.^82,83^

The monitoring of fermentation conditions confirmed the stability of the incubation environment, with the pH maintained between 5 and 6, and a well-balanced microbial composition. Microbiota composition exhibited some fluctuations in terms of relative abundance at the genus level among the different samples. These fluctuations were primarily influenced by the incubation time and appeared independent of the fermented substrate type. Both the incubation time and substrate type showed an influence on alpha diversity, suggesting a distinct microbial resilience related to the fermentation activity. Moreover, a putative relationship between phenolic catabolites and specific microbial genera that could be involved in (poly)­phenol catabolism was found. A limitation of these analyses could be the possible bias derived from the presence of fermentable dietary fibers in the growth medium, which can positively affect microbial composition by increasing the proportion of probiotics and other health-promoting bacteria.^84–86^ However, the presence of fiber is unlikely to affect the comparison among the substrates, as the same medium and fecal slurry were consistently used throughout the entire experiment.

Although additional *in vitro* experiments are needed to better elucidate the effect of PC_B2 on microbial composition in an *in vitro* environment, the enhancement of *in vitro* incubation with fecal slurries until 48 h allowed a better description of the microbial biotransformation and kinetics of colonic catabolites of these (poly)­phenols, reflecting a more physiological environment.

## Supplementary Material



## Notes

Chromatographic and spectrometric properties of the monitored parent flavan-3-ols and their gut microbial catabolites;^87^ concentrations of microbial catabolites obtained after 5, 8, 24, 30, and 48 h of fecal fermentation incubating (−)-EC, PC_B2, and PC_A2;^87^ filtering report of the samples; pH measures through each fermentation time (T0, T5, T8, T24, T30, and T48) in blank controls; and fermented samples for the substrates PC_A2, PC_B2, and (−)-EC (PDF)

## Abbreviations

EC: (−)-epicatechin
PC_B2: procyanidin B2
PC_A2: procyanidin A2
PCA: principal component analysis
PC: principal component
ASVs: amplicon sequence variants
PCoA: principal coordinates analysis
SCFAs: short-chain fatty acids
