# Supplementary material for: Optimization of an In Vitro Colonic Fermentation: Insights into Flavan-3-ol Catabolism and Microbiota Modulation
Source: J Agric Food Chem. 2025 Jun 18;73(26):16429–43. doi: 10.1021/acs.jafc.5c06932 (PMC12232328; doi:10.1021/acs.jafc.5c06932)
Supplement: Supplementary file 1 [file jf5c06932_si_001.pdf]

Optimization of an *in vitro* colonic fermentation: insights into flavan-3-ol catabolism and microbiota modulation

Nicole Tosi[1], *Leonardo Mancabelli*[2,3], *Giulia Alessandri*[4], *Francesca Turroni*[3,4], *Marco Ventura*[3,4], *Daniele Del Rio*[1,3], *Pedro Mena*[1,3], *Letizia Bresciani*[1,*]

[1] Human Nutrition Unit, Department of Food & Drug, University of Parma, Via Volturno 39, 43125, Parma, Italy

[2] Department of Medicine and Surgery, University of Parma, Via Gramsci 14, 43125, Parma, Italy

[3] Microbiome Research Hub, University of Parma, Parco Area delle Scienze 11A, 43124, Parma, Italy

[4] Laboratory of Probiogenomics, Department of Chemistry, Life Sciences, and Environmental Sustainability, University of Parma, Parco Area delle Scienze 11A, 43124 Parma, Italy

Supplementary Information (SI):

Supplementary Table S1. Chromatographic and spectrometric properties of the monitored parent flavan-3-ols and their gut microbiota catabolites. Nomenclature of catabolites is reported as proposed by Kay et al.[1] Abbreviations are reported under brackets. RT means retention time; *m/z* means mass to charge ratio; CE means collision energy; STD means standard used for quantification; n.d. means targeted but not detected; n.q. means detected but not quantified.

Supplementary Table S2. Concentrations (μmol/L) of microbial catabolites obtained after 5, 8, 24, 30 and 48 h of faecal fermentation incubating (−)-epicatechin (EC), procyanidin dimer B2 (PC_B2) and procyanidin dimer A2 (PC_A2). Data are expressed as mean ± SD (n=3). Different lower-case letters indicate significant differences comparing the same fermented substrate after different incubation periods (5, 8, 24, 30, 48 h) ($p < 0.05$). Different upper-case letters indicate significant differences among different fermented parent compounds (EC, PC_B2, PC_A2) considering the same incubation period ($p < 0.05$). Nomenclature of catabolites was based on Kay et al.[1]

Supplementary Table S3. Filtering report of the samples sequenced in this study.

Supplementary Figure S1. Composition of the faecal microbiota in a frozen faecal sample (faeces) and at each incubation time (T0, T8, T24, T30, T48) in blank controls (F) and in fermented samples for the substrates procyanidin dimer A2, procyanidin dimer B2 and (−)-epicatechin (EC). The composition is analyzed in terms of taxonomical classification in (A) phyla, (B) families and (C) genera.

Table S1. Chromatographic and spectrometric properties of the monitored parent flavan-3-ols and their gut microbiota catabolites. Nomenclature of catabolites is reported as proposed by Kay et al.[1] Abbreviations are reported under brackets. RT means retention time; *m/z* means mass to charge ratio; CE means collision energy; STD means standard used for quantification; n.d. means targeted but not detected; n.q. means detected but not quantified.

Compound (Abbreviation)	RT (min)	Parent ion (M - H)$^-$ (*m/z*)	S-lens	Quantifier		Qualifier		STD
				Product ion (*m/z*)	CE (V)	Product ion (*m/z*)	CE (V)	
**Parent compounds**								
Procyanidin dimer A2 (PC_A2)	4.85	575	131	289	25	449	25	PC_A2
Procyanidin dimer B2 (PC_B2)	4.12	577	131	289	27	407	25	PC_B2
(−)-Epicatechin (EC)	4.21	289	112	245	18	203	23	EC
**Fission catabolites**								
1 Fission Dimer A2 (1-fission A2)	5.10	577	131	577	25	291	25	PC A2
1 Fission Dimer B2 (1-fission B2)	4.73	579	131	291	25	289	25	PC B2
2 Fission Dimer A2 (2-fission A2)	-	579	-	-	-	-	-	n.d.
2 Fission Dimer B2 (2-fission B2)	-	581	131	581	25	291	25	n.d.
**Diphenylpropan-2-ol derivatives (DPPOLs)**								
1-(3′,4′-Dihydroxyphenyl)-3-(2″,4″,6″-trihydroxyphenyl)-propan-2-ol (3′,4′-DiOH-DPPOL)	4.21	291	98	123	30	247	16	3′,4′-diHPVL
1-(Hydroxyphenyl)-3-(2″,4″,6″-trihydroxyphenyl)-propan-2-ol (OH-DPPOL)	4.77	275	98	231	16	191	30	3′,4′-diHPVL
**Phenyl-γ-valerolactones (PVLs)**								
5-(3′,4′-Dihydroxyphenyl)-γ-valerolactone (3′,4′-DiHPVL)	4.25	207	81	163	18	122	21	3′,4′-diHPVL
5-(4′-Hydroxyphenyl)-γ-valerolactone (4′-HPVL)	-	191	67	147	16	106	31	n.d.
5-(3′-Hydroxyphenyl)-γ-valerolactone (3′-HPVL)	5.01	191	67	147	16	106	31	3′-HPVL
5-Phenyl-γ-valerolactone (PVL)	-	175	-	-	-	-	-	n.d.
**Phenylvaleric acids (PVAs)**								
4-Hydroxy-5-(3′,4′-Dihydroxyphenyl)valeric (4-OH-3′,4′-DiHPVA)	-	225	-	-	-	-	-	n.d.
5-(3′,4′-Dihydroxyphenyl)valeric acid (3′,4′-DiHPVA)	5.02	209	63	191	15	165	12	3′,4′-diHPVL
4-Hydroxy-5-(hydroxyphenyl)valeric acid (4-OH-HPVA)	4.30	209	63	147	20	101	20	3′,4′-diHPVL
5-(4′-Hydroxyphenyl)valeric acid (4′-HPVA)	-	193	71	147	30	175	30	n.d.
5-(3′-Hydroxyphenyl)valeric acid (3′-HPVA)	5.50	193	71	147	30	175	30	3′-HPVL
4-Hydroxy-5-(phenyl)valeric acid (4-OH-PVA)	5.41	193	-	-	-	-	-	n.d.
5-Phenylvaleric acid (PVA)	-	177	-	-	-	-	-	n.d.

**Phenylpropanoic acids (PPAs)**								
3-(4′-Hydroxy-3′-methoxyphenyl)propanoic acid (3′-MeO-4′-HPPA)	-	195	-	-	-	-	-	n.d.
3-(3′,4′-Dihydroxyphenyl)propanoic acid (3′,4′-DiHPPA)	3.16	181	64	137	14	109	18	n.d.
2-Hydroxy-3-(hydroxyphenyl)propanoic acid (2-OH-HPPA)	-	181	-	-	-	-	-	n.d.
3-(4′-Hydroxyphenyl)propanoic acid (4′-HPPA)	4.26	165	64	121	13	93	14	n.d.
3-(3′-Hydroxyphenyl)propanoic acid (3′-HPPA)	4.45	165	64	121	13	119	18	3′-HPPA
2-Hydroxy-3-(phenyl)propanoic acid (2-OH-PPA)	-	165	-	-	-	-	-	n.d.
3-Phenylpropanoic acid (PPA)	5.60	149	60	149	10	105	13	n.q.
**Phenylacetic acids (PAAs)**								
3’,4’-Dihydroxyphenylacetic acid (3′,4′-DiHPAA)	1.80	167	35	123	13	122	28	3′,4′-DiHPAA
4’-Hydroxyphenylacetic acid (4′-HPAA)	3.20	151	75	107	13	-	-	n.d.
3’-Hydroxyphenylacetic acid (3′-HPAA)	3.77	151	75	107	13	-	-	n.d.
Phenylacetic acid (PAA)	-	135	40	91	10	135	10	n.q.
**Benzoic acids (BAs)**								
3,4-Dihydroxybenzoic acid (3,4-DiHBA)	1.54	153	74	109	16	108	26	n.q.
4-Hydroxybenzoic acid (4-HBA)	2.58	137	72	93	16	-	-	4-HBA
3-Hydroxybenzoic acid (3-HBA)	3.63	137	72	93	16	-	-	n.d.
Benzoic acid (BA)	4.96	121	68	77	13	121	10	n.d.
**Benzaldehydes (BALs)**								
3,4-Dihydroxybenzaldehyde (3,4-DiHBAL)	2.30	137	72	136	22	108	26	n.d.
4-Hydroxybenzaldehyde (4-HBAL)	3.61	121	68	92	26	120	20	4-HBAL
**Benzyl alcohols (BOHs)**								
Hydroxybenzyl alcohol (OH-BOH)	-	123	-	-	-	-	-	n.d.
**Benzene derivatives (BZs)**								
Benzene-1,3,5-triol (1,3,5-TriOH-BZ)	0.85	125	62	125	10	57	17	n.d.
Benzene-1,2-diol (1,2-DiOH-BZ)	-	109	-	-	-	-	-	n.d.

Table S2. Concentrations (µmol/L) of microbial catabolites obtained after 5, 8, 24, 30 and 48 h of faecal fermentation incubating (−)-epicatechin (EC), procyanidin dimer B2 (PC_B2) and procyanidin dimer A2 (PC_A2). Data are expressed as mean ± SD (n=3). Different lower-case letters indicate significant differences comparing the same fermented substrate after different incubation periods (5, 8, 24, 30, 48 h) ($p < 0.05$). Different upper-case letters indicate significant differences among different fermented parent compounds (EC, PC_B2, PC_A2) considering the same incubation period ($p < 0.05$). Nomenclature of catabolites was based on Kay et al. [1].

		Substrates		
**Catabolites**	**Time (h)**	**EC**	**PC_B2**	**PC_A2**
(−)-Epicatechin (EC)	5	-	0.4±0.0	-
	8	-	-	-
	24	-	-	-
	30	-	-	-
	48	-	-	-
1 Fission dimer A2 (1-fission A2)	5	-	-	36.1±0.4 a
	8	-	-	37.4±1.3 a
	24	-	-	39.8±1.5 a
	30	-	-	27.1±3.6 b
	48	-	-	26.2±3.0 b
1 Fission dimer B2 (1-fission B2)	5	-	0.2±0.1 a	-
	8	-	0.2±0.0 b	-
	24	-	-	-
	30	-	-	-
	48	-	0.3±0.0 a	-
1-(3′,4′-Dihydroxyphenyl)-3-(2″,4″,6″-trihydroxyphenyl)-propan-2-ol (3′,4′-DiOH-DPPOL)	5	2.7±0.7 A	1.4±0.7 a A	-
	8	-	0.2±0.0 b	-
	24	-	-	-
	30	-	-	-
	48	-	-	-
1-(Hydroxyphenyl)-3-(2″,4″,6″-trihydroxyphenyl)-propan-2-ol (OH-DPPOL)	5	15.2±2.0 a A	10.9±1.2 a B	-
	8	1.4±0.1 b A	0.4±0.1 c B	-
	24	-	-	-
	30	-	-	-
	48	-	3.9±0.0 b	-
5-(3′,4′-Dihydroxyphenyl)-γ-valerolactone (3′,4′-DiHPVL)	5	27.1±4.4 a A	25.1±3.9 a A	0.3±0.0 c B
	8	30.2±1.9 a A	28.0±0.8 a A	0.4±0.0 c B
	24	24.4±1.2 a A	23.5±1.1 a A	0.6±0.0 b B
	30	14.7±1.0 b A	10.3±0.7 b B	0.7±0.1 ab C
	48	12.7±2.4 b A	9.9±0.0 b A	0.9±0.1 a B
5-(3′-Hydroxyphenyl)-γ-valerolactone (3′-HPVL)	5	12.2±1.9 c A	12.0±1.4 d A	-
	8	52.6±1.3 ab A	44.0±0.4 b B	-
	24	54.9±2.2 a A	50.0±3.7 a A	0.4±0.1 b B
	30	43.6±2.4 b A	29.3±0.2 c B	0.4±0.0 b C
	48	37.4±7.0 b A	14.2±0.0 d B	0.7±0.2 a C
5-(3′,4′-Dihydroxyphenyl)valeric acid (3′,4′-DiHPVA)	5	0.1±0.0 b	-	-
	8	0.2±0.0 a A	0.2±0.0 b A	-
	24	0.2±0.0 a A	0.3±0.0 a A	-
	30	0.2±0.0 a A	0.1±0.0 c B	-
	48	-	-	-

4-Hydroxy-5-(hydroxyphenyl)valeric acid (4-OH-HPVA)	5	1.2±0.2 c A	1.0±0.3 d A	-
	8	3.6±0.0 b A	2.9±0.2 b B	-
	24	6.3±0.5 a A	5.7±0.2 a A	-
	30	5.4±0.2 a A	2.8±0.3 b B	-
	48	3.5±0.6 b A	2.1±0.0 c B	-
5-(3′-Hydroxyphenyl)valeric acid (3′-HPVA)	5	-	-	-
	8	-	-	-
	24	1.1±0.0 a A	0.9±0.1 a B	-
	30	1.0±0.2 a A	0.7±0.1 b A	-
	48	1.1±0.2 a	-	-
3-(3′-Hydroxyphenyl)propanoic acid (3′-HPPA)	5	1.1±0.1 b C	1.6±0.2 b B	4.9±0.2 bc A
	8	2.4±0.4 a B	3.0±0.3 b B	6.8±0.7 ab A
	24	1.3±0.3 b B	1.1±0.4 b B	8.1±2.2 a A
	30	1.4±0.4 b B	3.2±1.1 b B	6.2±1.0 abc A
	48	1.8±0.1 ab C	10.5±0.0 a A	3.2±0.1 c B
2-(3′,4′-Dihydroxyphenyl)acetic acid (3′,4′-DiHPAA)	5	-	2.7±0.6 a	-
	8	-	1.8±0.3 b	-
	24	-	-	-
	30	-	-	-
	48	-	-	-
4-Hydroxybenzoic acid (4-HBA)	5	-	-	-
	8	-	-	-
	24	-	-	-
	30	1.9±0.5 a	-	-
	48	1.5±0.1 a A	0.5±0.0 B	0.4±0.1 B
4-Hydroxybenzaldehyde (4-HBAL)	5	-	-	-
	8	-	0.1±0.0 b	-
	24	-	0.3±0.0 a	-
	30	0.7±0.2 a	-	-
	48	0.3±0.1 b	-	-

Table S3. Filtering report of the samples sequenced in this study. The samples included a frozen fresh faecal sample (FT), blank controls (CTRL) at each fermentation time, and fermented samples at each fermentation time for the substrates procyanidin dimer A2 (PC_A2), procyanidin dimer B2 (PC_B2) and (−)-epicatechin (EC).

Sample	Time (h)	Input	Filtered	Denoised	Non-chimeric
**FT**	**-**	32965	31280	31280	27378
**CTRL**	**0**	52260	49103	49103	44237
**CTRL**	**8**	60748	57470	57470	50975
**CTRL**	**24**	53325	50523	50523	45030
**CTRL**	**30**	55874	53085	53085	47989
**CTRL**	**48**	33004	31204	31204	27855
**PC_A2**	**0**	45351	42653	42653	37991
**PC_A2**	**8**	42912	40213	40213	36217
**PC_A2**	**24**	41681	39198	39198	35013
**PC_A2**	**30**	57452	54084	54084	50611
**PC_A2**	**48**	25258	23816	23816	22186
**PC_B2**	**0**	44797	42631	42631	38363
**PC_B2**	**8**	43416	41207	41207	36610
**PC_B2**	**24**	48092	45907	45907	41485
**PC_B2**	**30**	61422	58207	58207	52265
**PC_B2**	**48**	50880	48398	48398	45633
**EC**	**0**	46581	44038	44038	39194
**EC**	**8**	57120	54168	54168	48719
**EC**	**24**	59271	56232	56232	51370
**EC**	**30**	40539	38040	38040	34030
**EC**	**48**	73260	69683	69683	61913

Figure S1. pH measures through each fermentation time (T0, T5, T8, T24, T30, T48) in blank controls (CTRL) and fermented samples for the substrates procyanidin dimer A2 (PC_A2), procyanidin dimer B2 (PC_B2) and (−)-epicatechin (EC).

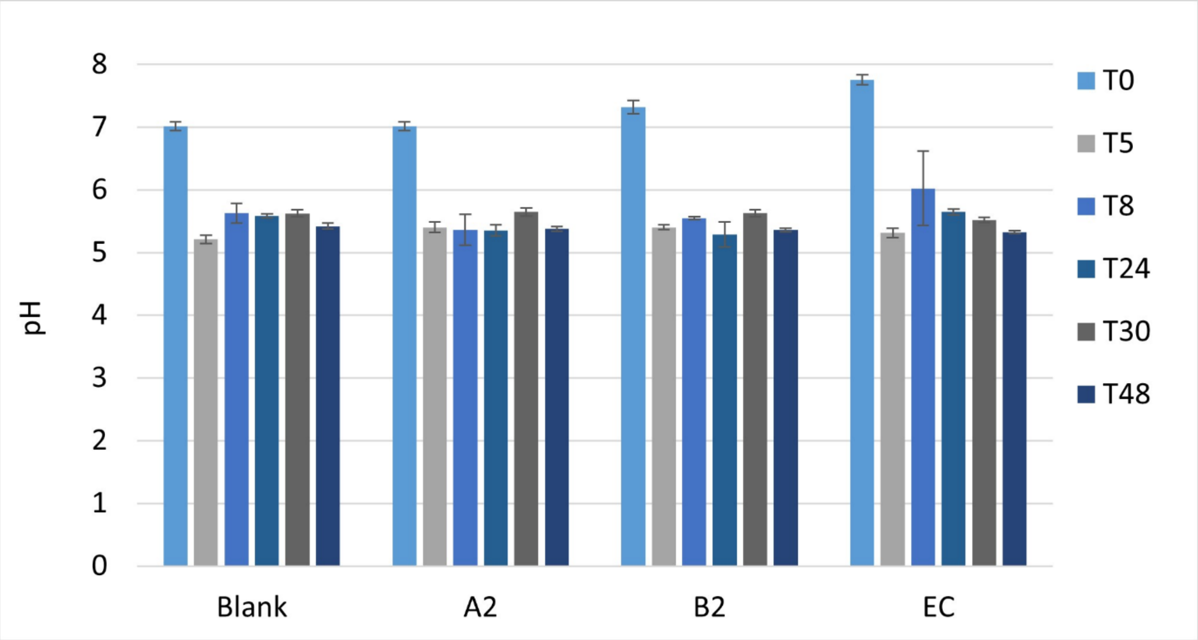

Supplementary References

(1) Kay, C. D.; Clifford, M. N.; Mena, P.; McDougall, G. J.; Andres-Lacueva, C.; Cassidy, A.; Del Rio, D.; Kuhnert, N.; Manach, C.; Pereira-Caro, G.; Rodriguez-Mateos, A.; Scalbert, A.; Tomás-Barberán, F.; Williamson, G.; Wishart, D. S.; Crozier, A. Recommendations for Standardizing Nomenclature for Dietary (Poly)Phenol Catabolites. *Am. J. Clin. Nutr.* **2020**, *112* (4), 1051–1068. https://doi.org/10.1093/ajcn/nqaa204
